# Overcoming pitfalls in multi-stack diffusion MRI for tractography reconstruction of skeletal muscles

**DOI:** 10.1038/s41598-026-63269-6

**Published:** 2026-07-22

**Authors:** Manuela Zimmer, Geoffrey Handsfield, Paul Condron, Samantha Holdsworth, Flavio Dell’Acqua, Filiz Ateş

**Affiliations:** 1 Experimental Biomechanics Group, Institute of Structural Mechanics and Dynamics in Aerospace Engineering, University of Stuttgart, Pfaffenwaldring 27, 70569 Stuttgart, Germany; 2 Auckland Bioengineering Institute, The University of Auckland, 70 Symonds Street, Auckland, 1010 New Zealand; 3 Department of Orthopaedics, University of North Carolina at Chapel Hill, 130 Mason Farm Rd Suite 3147, Chapel Hill, NC 27514 USA; 4 Lampe Joint Department of Biomedical Engineering, University of North Carolina at Chapel Hill, North Carolina State University, 333 South Columbia Street, Chapel Hill, NC 27514 USA; 5 Department of Anatomy and Medical Imaging, Faculty of Medical and Health Sciences & Centre for Brain Research, University of Auckland, 85 Park Road, Auckland, 1023 New Zealand; 6 Mātai Medical Research Institute, Childers Road 466, Tairāwhiti-Gisborne 4010, Gisborne, New Zealand; 7 Neuroimaging and Biophysics Lab (NBL), Department of Neuroimaging, Institute of Psychiatry, Psychology & Neuroscience, King’s College London, 16 De Crespigny Park, London, SE5 8AF UK

**Keywords:** Magnetic resonance imaging, Diffusion tensor imaging, Tractography, Skeletal muscle anatomy, Muscle fibre reconstruction, Anatomy, Biological techniques, Engineering, Medical research

## Abstract

Accurate in vivo characterization of skeletal muscle structure is essential for understanding muscle function, assessing pathologies, and developing musculoskeletal models. Magnetic resonance imaging (MRI) and diffusion MRI enable visualization of muscle morphology and fibre architecture in vivo, but imaging skeletal muscles often requires an extended field of view. At large distances from the magnet isocentre, gradient non-linearities and magnetic field inhomogeneities can cause severe image distortions. Here, we present multi-stack structural and diffusion-tensor echo-planar images of the lower limb using selected stack lengths and acquisition parameters to provide realistic examples. Geometric distortions, fractional anisotropy maps, and tractography-reconstructed fibre tracts are used to demonstrate anatomical plausibility and consistency across overlapping slices. In the presented examples, pronounced distortions, compromised fat suppression, and fibre reconstruction errors are observed under off-isocentre imaging conditions. While shorter stack lengths may help reduce distortions, our examples also indicate that factors such as shimming strategy, participant positioning, and post-processing can influence image quality. Our work highlights key pitfalls in muscle diffusion MRI, providing practical guidance grounded in MRI physics to support the design of multi-stack MRI protocols. These considerations support more reliable multi-stack diffusion MRI for musculoskeletal research and biomechanical modelling.

**Supplementary Information:**

The online version contains supplementary material available at 10.1038/s41598-026-63269-6.

## Introduction

Characterization of muscle function under varying physiological and pathological conditions is a central focus in biomechanics. Because muscle structure and function are tightly linked, accurately characterizing in vivo morphology and fibre organization is essential for studying their mechanical behaviour. Computational three-dimensional (3D) muscle models, such as finite-element approaches ^1, 2^, offer a controlled in silico framework to investigate aspects of muscle behaviour that are difficult to assess experimentally. Yet, only when grounded in realistic anatomy and accurate description of tissue behaviour, can such models capture the structural-functional coupling that governs muscle performance.

Magnetic resonance imaging (MRI) enables precise visualization of muscle morphology ^3^, and diffusion MRI with tractography algorithms (e.g., ^4^) allow the reconstruction of 3D muscle fibre architecture in vivo ^5, 6^. However, the integration of diffusion MRI-based fibre architecture into finite-element models (e.g., ^7^) remains limited, and alternative solutions such as pennation angle values from literature ^8, 9^ or fluid flow simulations ^10, 11^ are used. The limited application of diffusion MRI may, in part, reflect the lack of standardized MRI protocols ^12^ and the challenge of validating in vivo muscle fibre reconstructions. In practice, the accuracy of in vivo diffusion MRI-based muscle fibre reconstructions is generally unknown, so researchers must rely on achieving sufficient image quality to obtain anatomically plausible results.

Although several research groups have offered guidance on image acquisition and analysis for skeletal muscle diffusion MRI (e.g., ^6, 13, 14^), one challenge remains overlooked: the extended field-of-view (FOV) required to capture the whole muscle from origin to insertion introduces systematic imaging errors that are not well characterized in skeletal muscle diffusion MRI. MRI relies on three types of magnetic fields: the main magnetic field (B_0_), the radiofrequency field, and the gradient fields. Imperfections in these fields, i.e., field inhomogeneities and gradient non-linearities, specifically at large distances from the isocentre, lead to geometrical distortions ^15^ and bias in diffusion tensor estimation ^16^. Despite the use of correction methods ^17, 18, 19^, the large FOV in muscle diffusion MRI may extend into regions where these techniques no longer fully compensate for magnetic field imperfections. Echo-planar imaging (EPI ^20^, which is the acquisition strategy commonly used for muscle diffusion MRI ^13)^, is especially sensitive to field inhomogeneities and susceptibility artefacts due to the long readout times. In addition, eddy currents from strong, rapidly switching diffusion-sensitizing gradients can interfere with the B_0_ field ^21^. Taken together, these factors make muscle diffusion MRI inherently susceptible to artefacts arising from magnetic field imperfections.

Dividing the imaging volume into multiple stacks with table repositioning reduces the FOV for each stack and therefore mitigates adverse off-isocentre effects. However, this approach introduces new challenges: in multi-stack acquisitions, adjacent stacks are acquired at different spatial positions relative to the magnet isocentre, such that even small inconsistencies in magnetic field conditions can result in misalignment of anatomical structures and discontinuities in derived fibre trajectories. Consequently, the choice of stack length becomes a critical yet largely unexamined parameter, and its impact on spatial accuracy and tractography reliability in skeletal muscle diffusion MRI has not been evaluated. As a result, it remains unclear how acquisition parameters, particularly stack length, participant positioning, and correction strategies, interact to influence spatial consistency and fibre reconstruction accuracy in multi-stack muscle diffusion MRI.

In this work, we present illustrative examples of spatial distortions and tractography reliability in multi-stack skeletal muscle diffusion MRI using representative lower leg images acquired with different acquisition parameters, stack lengths, and correction strategies. We examined geometrical distortions, image alignment, diffusion MRI-derived metrics, and tractography-based fibre reconstructions across configurations to illustrate how acquisition and post-processing choices influence the quality of multi-stack muscle diffusion MRI data. This work aims to provide practical, physics-informed guidance based on representative examples to support the implementation of large-FOV multi-stack muscle diffusion MRI and improve fibre reconstruction reliability. By linking acquisition-related pitfalls to their downstream effects on tractography-based fibre reconstruction of whole muscles, it offers actionable considerations for protocol design in musculoskeletal applications.

## Results

### Bilateral scans

**Fig. 1:**
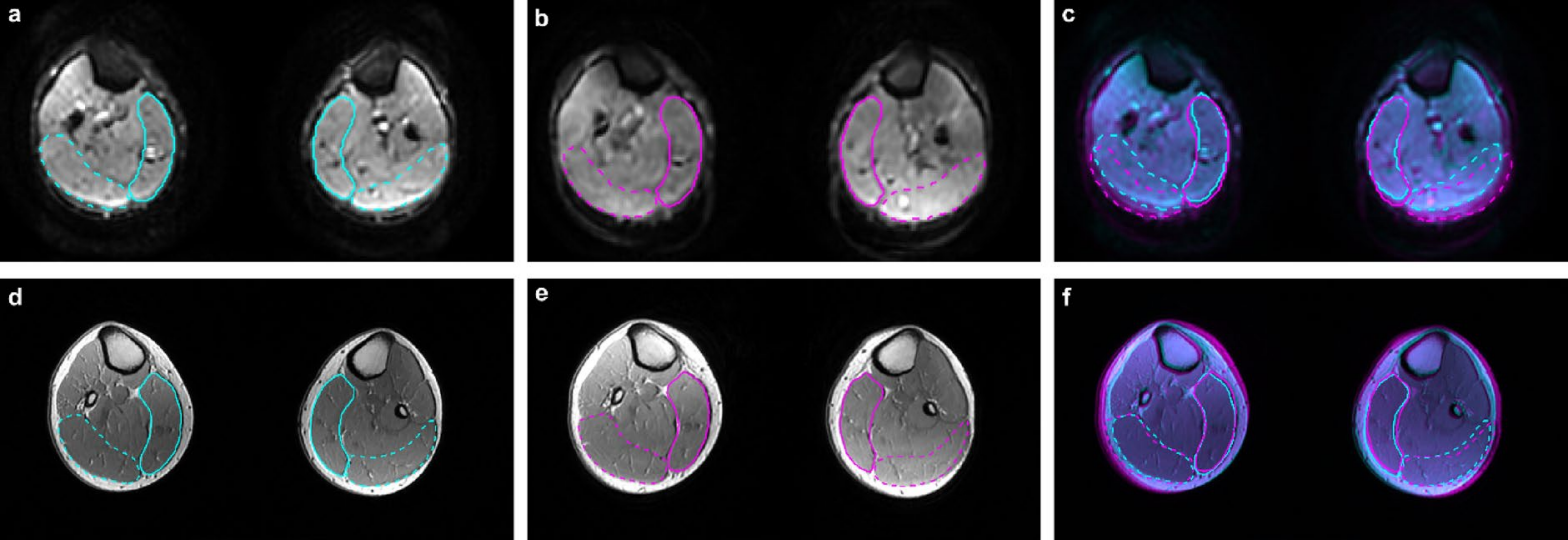
Spatial distortion in magnetic resonance images acquired off-isocentre. Axial echo-planar imaging (**a**-**c**) and 3D gradient echo (**d**-**f**) images were acquired in a bilateral setup. The gastrocnemius medialis and lateralis muscles are outlined with a solid and dashed line, respectively. Images (**a**) and (**d**) show the slice positioned at the isocentre, while in images (**b**) and (**e**) the slice was located at 14.4 cm off-isocentre. The overlays (**c**, **f**) demonstrate substantial spatial distortion [(**a**, **d**) in cyan; (**b**, **e**) in magenta]. Images were taken from a 37-year-old man (body weight and height: 76 kg, 182 cm). Image settings are listed in Table S1 (IDs 1 A, 1B, 2 A, and 2B).

A comparison of bilateral diffusion-tensor EPI (DT-EPI) and 3D gradient echo (3D-GRE) images acquired at the isocentre and at 14.4 cm off-isocentre demonstrated visible spatial differences (Fig. 1). The maximum radial distance of the limbs in the slice located at the isocentre was $\:{R}_{max}$ = 169.2 mm, while it was $\:{R}_{max}$ = 225.1 mm for the slice located 14.4 cm off-isocentre. The Dice similarity coefficient (DSC) of the gastrocnemii muscle masks obtained from the two images (isocentre vs. off-isocentre) was 0.905 for the 3D-GRE images and 0.770 for the EPI images.

**Fig. 2:**
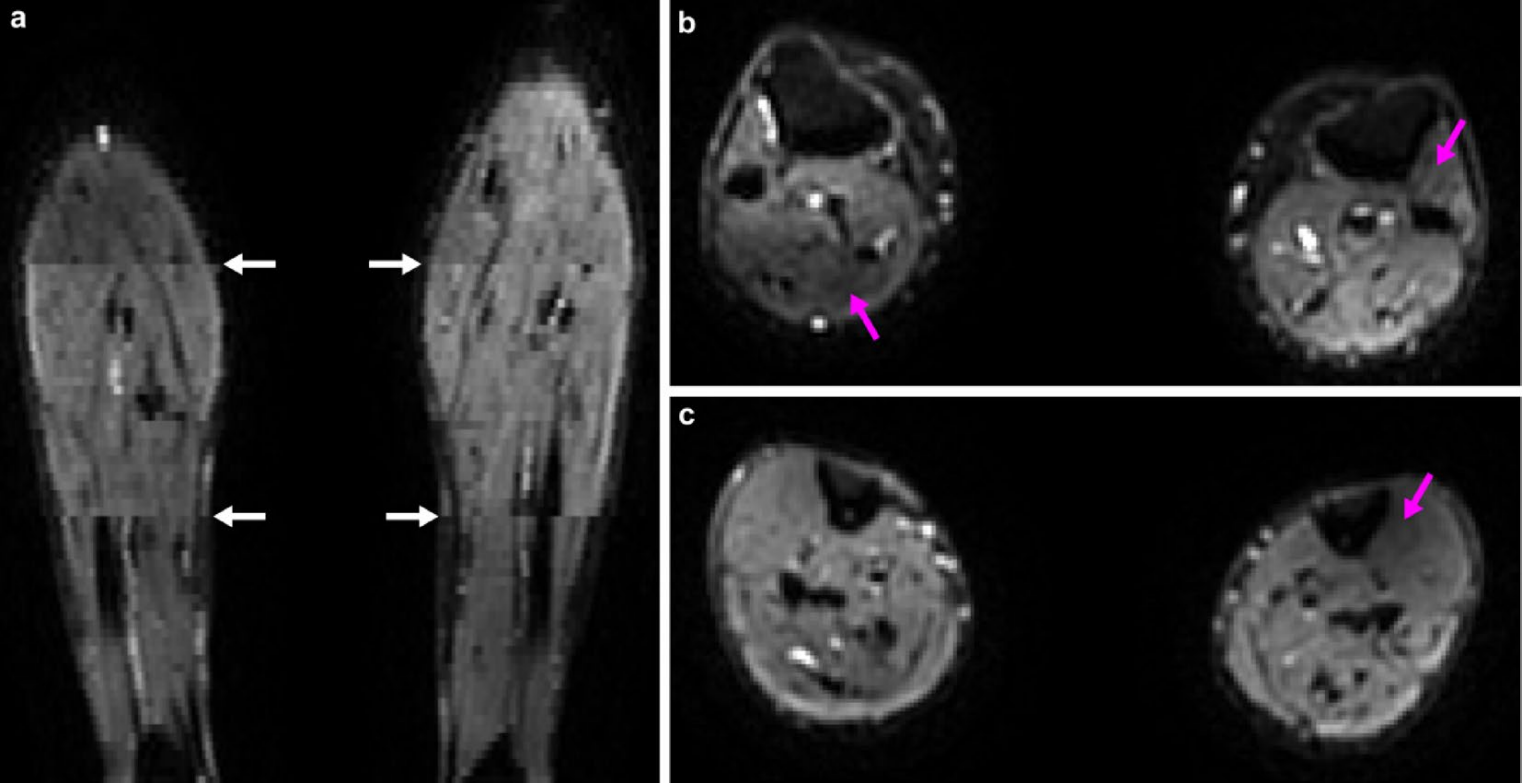
Spatial distortion and poor signal quality in bilateral images of a three-stack acquisition with 13.6 cm stack length. Misalignment of anatomical structures across stack boundaries (white arrows) is visible in images obtained from of echo-planar imaging (EPI) in coronal view (**a**). Axial images (**b**, **c**) represent the centre slice of the first two stacks and demonstrate areas of low signal intensity (magenta arrows). Images were taken from a 42-year-old man (body weight and height: 66 kg, 166 cm). Image settings are listed in Table S1 (ID 3).

A bilateral three-stack DT-EPI acquisition with 13.6 cm stack length demonstrated pronounced spatial distortion at the stack peripheries (Fig. 2a) and areas of low signal intensity and banding effects (Fig. 2b, c). The maximum radial distances of the limbs for each stack were $\:{R}_{max}$ = 179.2, 180.9, and 164.9 mm.

**Fig. 3:**
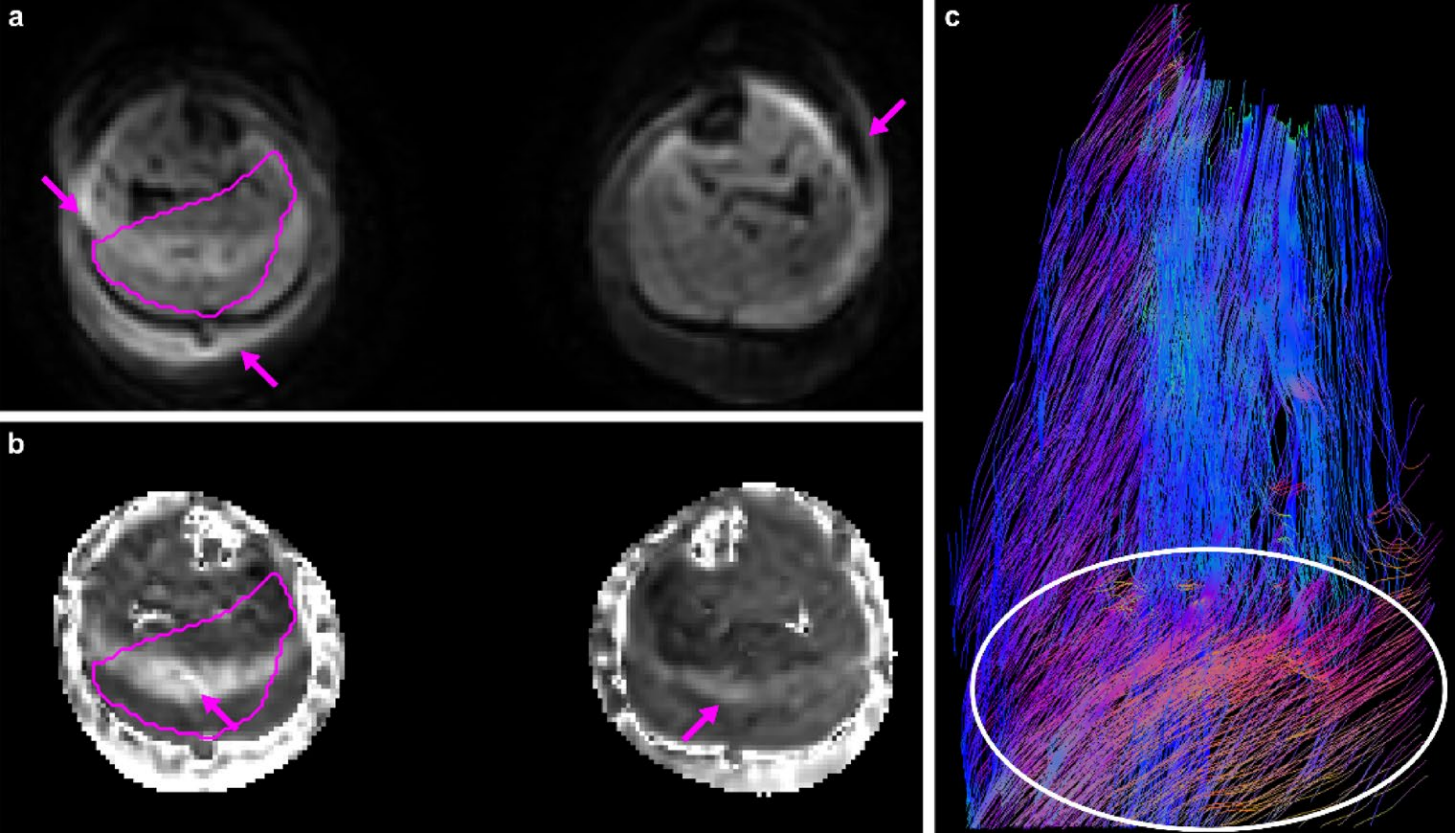
Fat ghosting artefacts in bilateral diffusion-tensor echo-planar images with 22.4 cm stack length. Axial diffusion-weighted echo-planar images (**a**) demonstrate fat ghosting at the stack periphery (slice 2/56), resulting in artefacts in the fractional anisotropy map (**b**). The affected area corresponds to the central part of the right soleus muscle (magenta outline), resulting in anatomically unrealistic tractography-based fibre reconstructions (**c**). Fibre tracts are visualised using RGB direction-encoding (red = left–right, green = anterior–posterior, blue = superior–inferior). Images were taken from a 25-year-old woman (body weight and height: 60 kg, 164 cm). Image settings are listed in Table S1 (ID 4).

Bilateral DT-EPI images with 22.4 cm stack length and a maximal radial distance of $\:{R}_{max}$ = 216.2 mm demonstrated insufficient fat suppression at the stack periphery, which produced visible fat ghosting affecting the region of the soleus muscle (Fig. 3a). This artefact propagated into the fractional anisotropy (FA) maps (Fig. 3b) and resulted in tractography reconstructions of the soleus fibre architecture exhibiting abrupt orientation changes that deviate from expected anatomy (Fig. 3c).

### Unilateral scans

**Fig. 4:**
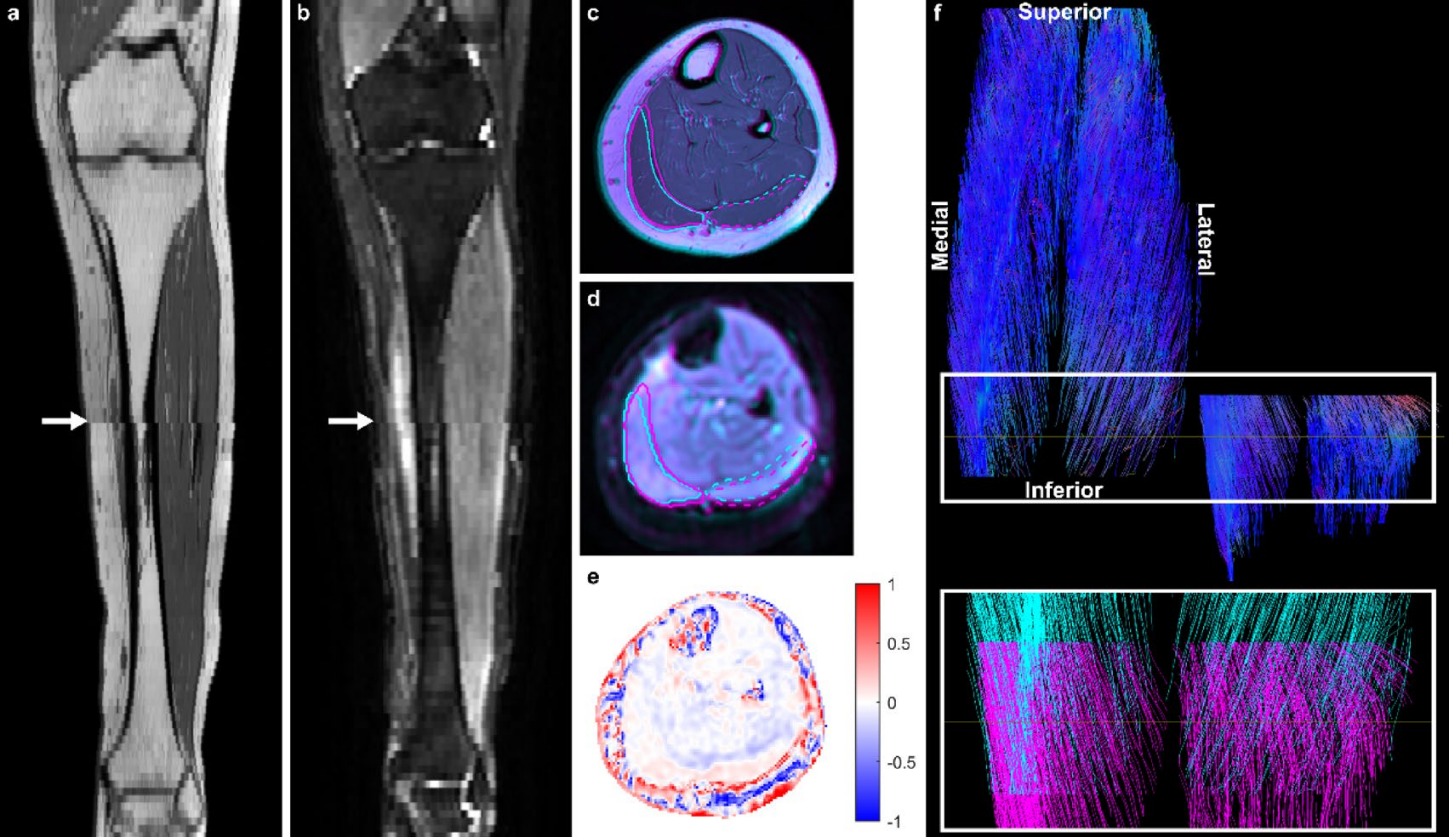
Adverse off-isocentre effects in a two-stack acquisition with 24 cm stack length. Fast-spin echo (FSE; **a**) and echo-planar imaging (EPI; **b**) images demonstrate misalignment of anatomical structures across stack boundaries (arrows) in coronal view. Axial FSE (**c**) and EPI (**d**) images represent overlapping slices with slice 4/60 from stack 1 (cyan) and slice 37/60 from stack 2 (magenta). Substantial spatial distortions are present and gastrocnemius medialis (solid line) and lateralis (dashed line) masks disagree between stacks. The difference in the fractional anisotropy map is shown in (**e**). Tractography reconstructions of the gastrocnemii muscles (**f**) differ across stacks. Fibre tracts from the individual stacks are either visualised using RGB direction-encoding (red = left–right, green = anterior–posterior, blue = superior–inferior) or overlaid in cyan (stack 1) and magenta (stack 2). Images were taken from a 25-year-old woman (body weight and height: 60 kg, 164 cm). Image settings are listed in Table S1 (IDs 5 A and 5B).

Unilateral two-stack DT-EPI and fast-spin echo (FSE) images with 24 cm stack length exhibited pronounced misalignment of anatomical features across stack boundaries (Fig. 4a, b). The maximum radial distances of the limbs for each stack were $\:{R}_{max}$ = 153.6 and 155.9 mm. Overlays of overlapping slices showed visible spatial distortions (Fig. 4c, d). The DSC of the gastrocnemii muscle masks evaluated from the overlapping slices was 0.854 for the FSE images and 0.797 for the EPI images. FA maps differed across the overlapping slices (Fig. 4e) with an average root-mean-squared error (RMSE) of 0.070 within the gastrocnemii muscle masks. Tractography reconstructions differed substantially between stacks (Fig. 4f).

**Fig. 5:**
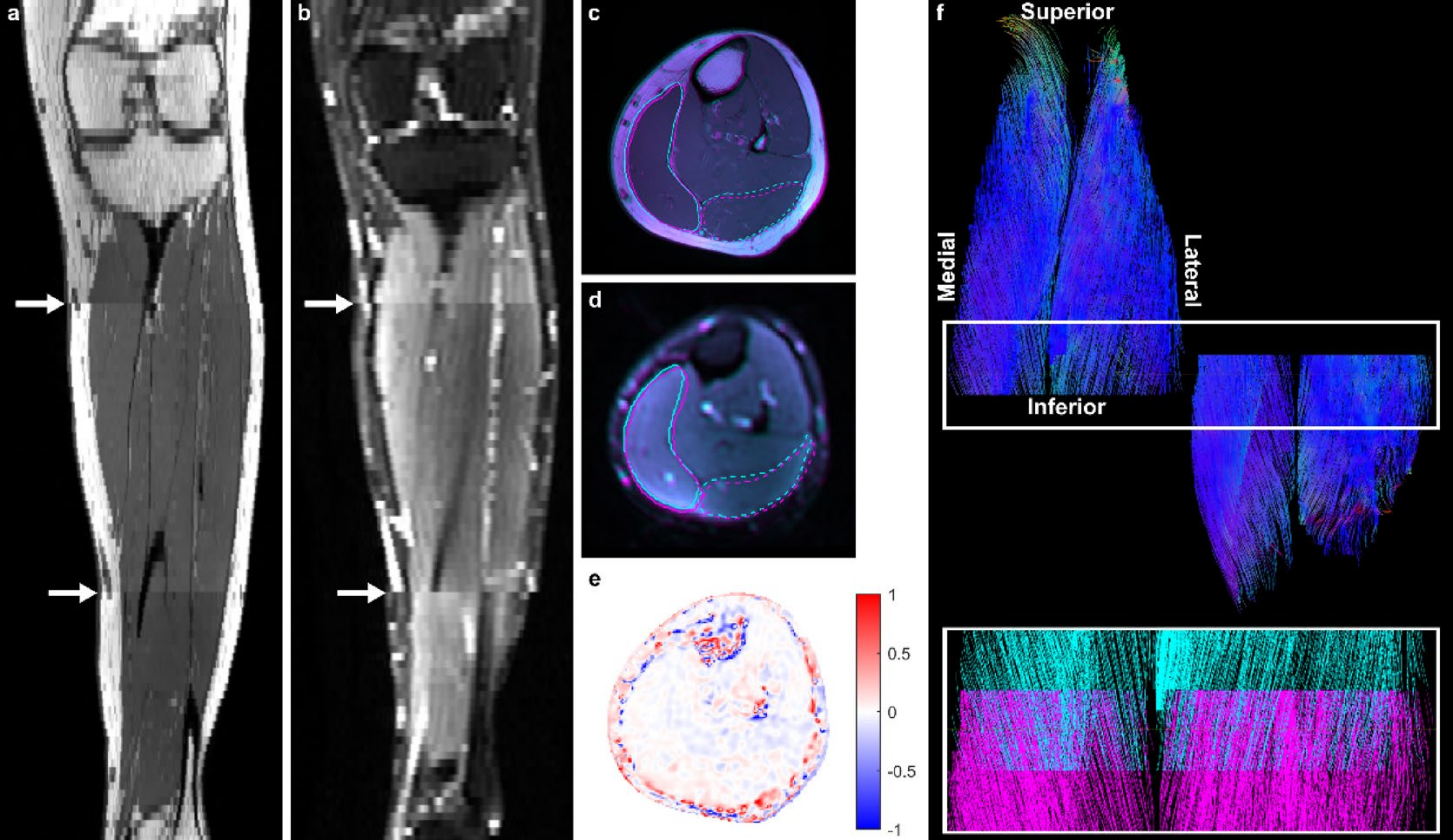
Reduced off-isocentre distortions in a three-stack acquisition with 16.8 cm stack length. Fast-spin echo (FSE; **a**) and echo-planar imaging (EPI; **b**) images demonstrate minor misalignment of anatomical structures across stack boundaries (arrows) in coronal view. Axial FSE (**c**) and EPI (**d**) images represent overlapping slices with slice 3/42 from stack 1 (cyan) and slice 41/42 from stack 2 (magenta). Minor spatial distortions are present and gastrocnemius medialis (solid line) and lateralis (dashed line) masks align reasonably between stacks. The difference in the fractional anisotropy map is shown in (**e**). Tractography reconstructions of the gastrocnemii muscles (**f**) differ across stacks. Fibre tracts from the individual stacks are either visualised using RGB direction-encoding (red = left–right, green = anterior–posterior, blue = superior–inferior) or overlaid in cyan (stack 1) and magenta (stack 2). Images were taken from a 22-year-old man (body weight and height: 80 kg, 179 cm). Image settings are listed in Table S1 (IDs 6 A and 6B).

In a unilateral three-stack acquisition with 16.8 cm stack length, anatomical structures were more closely aligned across stack boundaries (Fig. 5a, b). The maximum radial distances of the limbs for each stack were $\:{R}_{max}$ = 143.4, 143.5, and 111.0 mm. Marginal distortion in overlapping slices was observed (Fig. 5c, d). The DSC of the gastrocnemii muscle masks evaluated from the overlapping slices was 0.920 for the FSE images and 0.900 for the EPI images. Tractography reconstructions appeared more similar across stacks (Fig. 5f), and only small variations were observed in FA values across the overlapping slices (Fig. 5e) with an average RMSE of 0.046 within the gastrocnemii muscle masks.

**Fig. 6:**
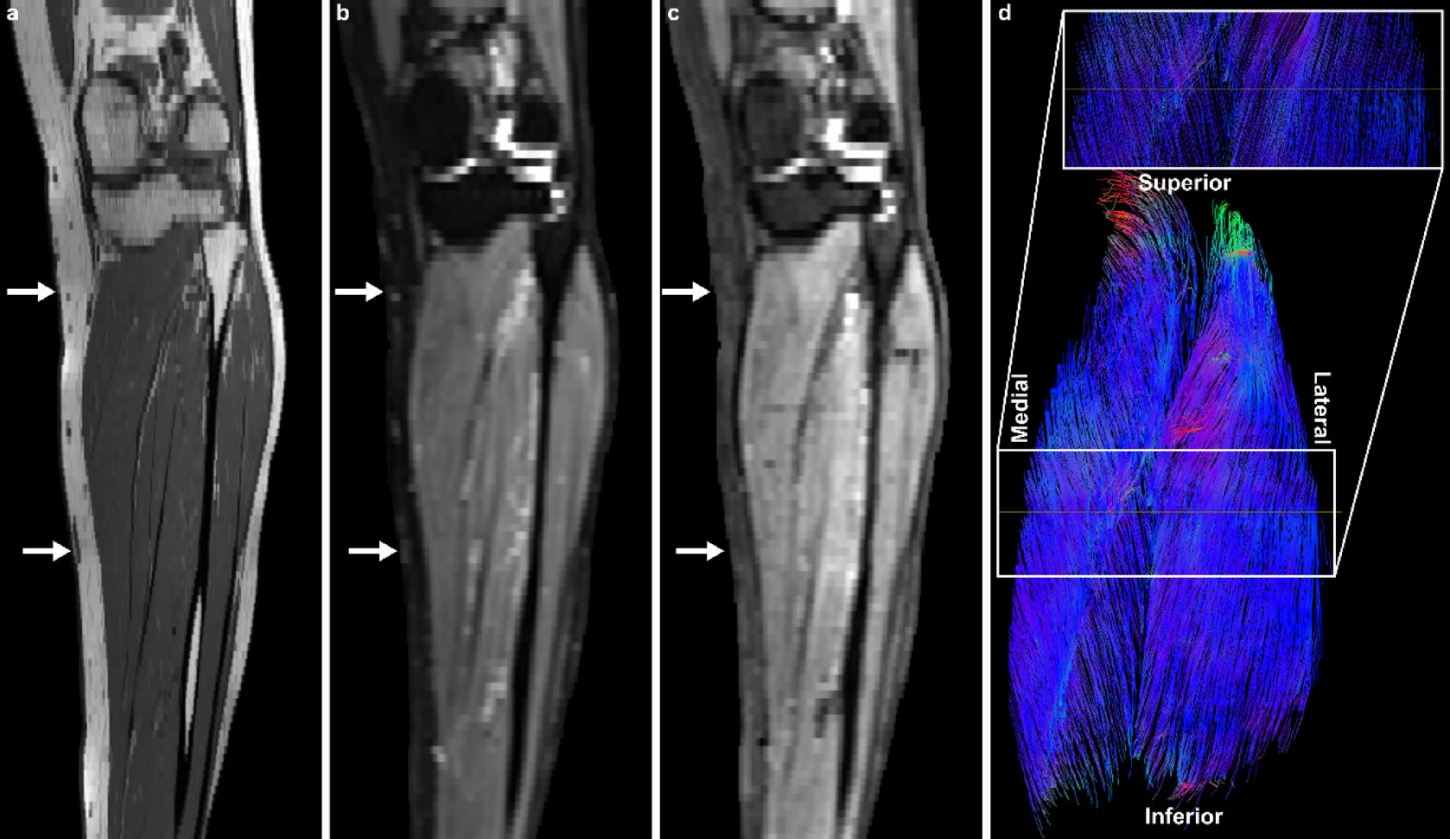
Three-stack acquisition with 15.2 cm stack length concatenated to a continuous volume. Fast-spin echo (FSE) and diffusion-tensor echo-planar imaging (DT-EPI) sequences were acquired with higher-order active shimming and real-time centre frequency correction. Stacks were concatenated to a continuous volume using weighted averaging of overlapping slices and intensity scaling across stacks. Excellent alignment of anatomical structures across the original stack boundary (arrows) of FSE (**a**), echo-planar images (no diffusion-weighting; (**b**), and DT-EPI images (**c**) is visible in coronal view. Continuous and anatomically feasible tractography-based fibre reconstructions were obtained from the gastrocnemii muscles (**d**). The magnification shows the original stack boundary. Fibre tracts are visualised using RGB direction-encoding (red = left–right, green = anterior–posterior, blue = superior–inferior). Images were taken from a 28-year-old woman (body weight and height: 75 kg, 175 cm). Image settings are listed in Table S1 (IDs 7 A, 7B, and 7 C).

Unilateral three-stack DT-EPI and FSE images with 15.2 cm stack length acquired with higher-order active shimming and real-time B_0_ frequency correction demonstrated spatial alignment across stack boundaries. The maximum radial distances of the limbs for each stack were $\:{R}_{max}$ = 135.8, 135.5, and 134.7 mm. Marginal distortions in overlapping slices were observed with a DSC of the gastrocnemii muscle masks of 0.942 for the FSE images and 0.936 for the EPI images (calculated from EPI images with phase-encode direction anterior-posterior). Minimal differences in FA values of overlapping slices were present with an average RMSE of 0.043 within the gastrocnemii muscle masks. Comparing the DT-EPI images with reversed phase-encoding direction revealed susceptibility-induced spatial distortions and chemical fat shift from the subcutaneous fat layer along the phase-encode direction (Supplementary Figure S1). Motion artefacts and eddy current distortions were minimal (Supplementary Figure S2). Susceptibility-induced distortion correction, as well as eddy current and motion correction, successfully unwarped the present distortions. Concatenating the three-stack acquisition to a continuous volume using weighted averaging of overlapping slices and intensity scaling across stacks resulted in well-aligned anatomical features across the original stack boundaries (Fig. 6a–c). Tractography performed on the continuous DT-EPI volume resulted in smooth and continuous fibre reconstructions of the gastrocnemii muscles across the original stack boundaries (Fig. 6d).

## Discussion

The presented lower limb images and tractography-based muscle fibre reconstructions highlight several pitfalls in multi-stack muscle diffusion MRI that can compromise image quality and result in geometrical distortions and discontinuities in reconstructed fibre trajectories. These illustrative observations suggest that multi-stack acquisitions, while necessary for imaging large fields of view, introduce specific challenges associated with off-isocentre imaging conditions that may affect spatial consistency across stacks.

### How stack length affects off-isocentre distortions

Our results indicate that imaging at large distances from the isocentre can be associated with visible image distortions (Fig. 1). These observations are in agreement with prior studies describing gradient nonlinearities, which arise from limitations in gradient coil design ^17, 18^, as a source of geometric distortions in the magnitude of several millimetres around 6–9 cm away from isocentre ^22^. Gradient nonlinearities have also been reported to cause intensity variations, voxel shifts, and variations in slice thickness across the imaging volume ^18, 23^, as well as to affect the magnitude and direction of the diffusion-sensitizing gradients in diffusion MRI ^19, 24^. In multi-stack acquisitions with table repositioning for each stack, these effects become particularly relevant at the stack boundaries, where adjacent stacks are acquired at different bore positions, meaning the same anatomical region can fall into areas where the magnetic field inhomogeneity and gradient nonlinearity differ. In diffusion MRI, resulting bias in the diffusion tensor can lead to disagreement of tractography-based fibre reconstructions spanning subsequent stacks (Fig. 4). As multi-stack approaches with sufficiently short stack length and resulting radial distance to the isocentre can mitigate the adverse effects of off-isocentre distortions (Figs. 5 and 6), stack length becomes a critical parameter. Previous studies using a multi-stack approach demonstrate a wide range of stack lengths spanning from 6.6 cm to 22.75 cm ^25, 26, 27, 28, 29, 30, 31, 32^. However, the impact of stack length on the image quality has received little attention. In some studies, stack length is even unreported (e.g., ^33,34,35)^ or images were acquired in a single large stack ^36^.

In the acquisitions examined here, shorter stack lengths were associated with reduced off-isocentre distortions, indicating that stack length can substantially influence visible off-isocentre distortions in large-FOV acquisitions. However, as acquisition parameters were not varied in isolation, this association should be interpreted cautiously, and the observed reduction in distortions cannot be attributed solely to stack length. Moreover, the optimal stack length is expected to depend on scanner- and protocol-specific factors. Identification of geometrical distortions without an undistorted reference image is challenging, making it difficult to assess the image quality retrospectively. Overlapping slices between stacks of a multi-stack acquisition can help reveal off-isocentre distortions: when anatomical structures, fibre reconstructions, and anisotropy measures align across the overlap, it suggests that inconsistencies at the stack periphery are small for the chosen configuration. As off-isocentre distortions may vary between vendors, scanners, and specificities of the imaging sequence, we recommend pilot imaging with varying stack length to identify the stack length that results in negligible off-isocentre distortions from the overlapping slices.

Furthermore, our images indicate that even image stacks with relatively short stack lengths (~ 15–16 cm) may benefit from higher-order active shimming and real-time B_0_ frequency correction, suggesting that field inhomogeneities should not be neglected even for short stacks. Field mapping techniques ^37^ or gradient nonlinear correction methods (e.g., ^19, 38)^, although not presented in this work, may have similar effects.

### How limb selection and participant positioning affect image quality

The difference in observed geometrical distortions between bilateral (Fig. 2) and unilateral (Fig. 5) scans illustrates that in-plane positioning of the region of interest can also affects off-isocentre distortions. In a bilateral setup, both legs are located at a lateral offset to the magnet centre, placing slices at larger radial distances from the isocentre [Eq. (3)]. Under the assumption that magnetic field imperfections scale with radial distance to the isocentre, a larger in-plane FOV requires shorter stack lengths to remain within the same amount of field imperfections. Therefore, we recommend reducing in-plane offsets as best as possible. For example, posterior offsets can be reduced by elevating the limb to the scanner centreline; lateral offsets in bilateral scans can be reduced by minimizing the gap between the limbs.

Bilateral scans may also demonstrate reduced signal uniformity and inconsistent fat suppression ^39^, which may indirectly affect the diffusion tensor estimation in diffusion MRI ^40^. Strategies such as parallel transmit techniques, radiofrequency shimming, or dielectric pads were suggested as ways to reduce these artefacts ^39, 40^.

Furthermore, joint angles should be standardized, and soft tissue deformation avoided to facilitate biomechanical interpretation of muscle morphology and fibre architecture. Custom support devices (e.g., ^34, 35, 41^) can be used to ensure consistent joint positioning and provide stability. Such stabilization also reduces inadvertent muscle activation, thereby minimizing activation-related changes in fibre architecture ^42^ and mitigating motion-related signal dropout ^43^.

### How DT-EPI-specific artefacts impact diffusion MRI quality

Besides B_0_ inhomogeneities arising from system design, patient-induced susceptibility differences ^44^ or eddy currents ^21^ can also affect field homogeneity. Moreover, DT-EPI images of the lower limb muscles may suffer from chemical fat shift of the subcutaneous fat layer along the phase-encode direction affecting superficial muscles. While geometric distortion can be corrected during post-processing ^45^, we recommend selecting the phase-encoding direction such that muscles of interest remain unaffected by the chemical fat shift, e.g., anterior-to-posterior phase encoding for posterior leg muscles.

Motion artefacts and eddy current distortions were minimal in the presented images with the moderate diffusion weighting (b-value of 500 s∙mm^− 2^) and small number of diffusion directions used. Nevertheless, we recommend incorporating respective corrections to the post-processing pipeline (e.g., in combination with susceptibility-induced distortion correction using *FSL*’s *eddy* tool ^21)^.

### How insufficient fat suppression can affect fibre reconstructions

Our images suggest that inadequate fat suppression (e.g., ^46^) can compromise the accuracy of diffusion MRI-derived muscle architecture (Fig. 3). Thus, effective fat suppression is essential for reliable tractography-based fibre reconstructions, but its performance can be compromised by reduced field homogeneity in larger FOVs. For example, chemical fat saturation methods employ a frequency-selective pulse, but field inhomogeneity shifts the fat resonance frequency. Similarly, slice-selective gradient reversal methods rely on a predictable off-resonance frequency between fat and water, yet B_0_ inhomogeneity introduces unpredictable fat shifts. Therefore, we recommend improving field homogeneity and selecting fat suppression methods that are less sensitive to field inhomogeneities, e.g., chemical fat saturation with a larger bandwidth. Specific implementation must be guided by scanner‑ and protocol‑specific constraints; however, the underlying physics considerations apply across vendors and can be used to guide fat suppression choices when configuring new protocols.

### How multi-stack diffusion MRI acquisitions can be processed as a continuous volume

Tractography-based fibre reconstructions performed on individual stacks inevitably lead to discontinuities at the stack boundaries, as tractography does not resolve individual muscle fascicles ^13^ and therefore cannot inherently establish fibre continuity between adjacent stacks. Thus, tractography performed on individual stacks may be inadequate for consistent whole-muscle reconstruction. We therefore recommend concatenating stacks into a continuous volume, which yields uninterrupted fibre trajectories (Fig. 6). Here, we applied weighted averaging of overlapping slices between adjacent stacks, assigning greater weights to slices closer to the isocentre. This approach was motivated by the expectation that magnetic field imperfections and associated distortions, increase with radial distance from the isocentre. Therefore, slices closer to the isocentre are ‘trusted’ more by applying a greater weight. We also applied an intensity scaling to compensate any differences in coil sensitivities. These steps are grounded in MRI physics considerations and are intended to mitigate residual technical differences between adjacent stacks and improve continuity of reconstructed fibre trajectories across stack boundaries. While the present work does not provide a systematic quantitative evaluation of the concatenation strategy, the approach represents a practically motivated method for handling multi-stack muscle diffusion MRI data. Dedicated phantom-based validation studies would be valuable in future work. Lastly, synthetic regeneration of DT-EPI images [Eq. (1)] was necessary prior to concatenation, as the diffusion gradient directions were altered by the applied motion and eddy-current correction ^21^, resulting in differing diffusion gradient tables across stacks. This can also occur during image registration, e.g., when aligning images that were acquired from different sequences (e.g., ^25, 28^) or when correcting for sequence-specific distortions (e.g., ^27, 34^). A limited number of diffusion-encoding directions, particularly in low signal-to-noise ratio conditions, can lead to instability in diffusion tensor estimation. This instability may propagate into synthesized DT-EPI images, potentially introducing errors in subsequent tractography-based fibre reconstructions. To mitigate these effects, we recommend acquiring a greater number of diffusion directions (i.e., more than six) and/or increasing voxel size to improve signal-to-noise ratio, provided that acquisition time constraints and spatial resolution requirements are adequately balanced.

Note that when applying spatial transformations voxels may be remapped outside the original image boundaries. This can result in empty voxels at the edges of the imaging volume, which may lead to partial loss of anatomical coverage. For multi-stack acquisitions without overlapping slices, this would result in ‘missing’ slices between stacks. To mitigate this, we recommend adding empty slices (‘zero-padding’) prior to transformation, which preserves spatial coverage in the slice direction.

### Limitations and future directions

This work is intended as an illustrative, technical contribution that provides practice-oriented guidance rather than a controlled experimental or optimisation study. The presented examples were acquired under varying acquisition and processing conditions, with multiple factors changing concurrently. As a result, the individual contribution of specific parameters cannot be isolated, and the observations should be interpreted as descriptive examples rather than evidence of independent causal effects.

All images presented were automatically up-sampled from the scanner during reconstruction. While this can be beneficial for visual inspection or segmentation of anatomical structures, it can hinder post-processing tools such as denoising ^47, 48, 49^ or motion and eddy current correction ^21^. Note that some reconstruction methods, e.g., GE Healthcare’s AIR™ Recon DL, may not allow setting specific reconstruction matrix sizes, i.e., disabling up-sampling.

Some of the DT-EPI datasets presented in this study were acquired with only six diffusion-encoding directions, which may compromise the robustness of diffusion tensor estimation and influence derived measures, including FA and tractography results. Notably, the primary purpose of these acquisitions was to obtain reversed phase-encode DT-EPI images for susceptibility-induced distortion correction, for which diffusion encoding is not required. However, when the data are post-processed and subsequently used for tractography, inaccuracies in diffusion tensor estimation may propagate and manifest as erroneous fibre reconstructions.

Imaging large muscles, such as those in the lower leg, requires a receiver coil that covers at least half of the limb. In practice, this is typically achieved using large flexible coils, as repositioning smaller coils between scans can introduce participant movement and misregistration ^26^. However, flexible coils may have fewer receiver channels and distinct sensitivity profiles compared to rigid coils. Consequently, caution is warranted when applying image acceleration techniques, such as parallel imaging and simultaneous multi-slice acquisition, together with soft coils. These methods rely on stable coil sensitivity profiles for accurate reconstruction, and sufficient coil sensitivity profiles along the slice direction are essential to distinguish signals from multiple slices ^50^. Additionally, any coil movement between the calibration phase and image acquisition may compromise the accuracy of coil sensitivity calibration. Moreover, the use of advanced reconstruction methods for accelerated acquisition may come at the cost of compromised image quality if not addressed adequately (e.g., ^51, 52^).

Although the presented images were all acquired using the same scanner, the derived considerations are expressed vendor-unspecific and are grounded in fundamental physics principles. Thus, we expect the qualitative principles to be applicable and beneficial across MRI systems, although their quantitative impact may differ and requires validation across vendors and implementations.

Quantitative metrics such as the DSC of segmentation masks or RMSE of FA maps are reported descriptively to support the visual observations presented. As such, these metrics should be interpreted within the technical scope of the study. For true geometric validation, phantom-based, landmark-based, or displacement-field measurements would be needed. Consistent with the illustrative nature of this work, the effects of individual parameters such as stack length, shimming strategy, and participant positioning were not systematically isolated. Systematic evaluation of individual imaging parameters remains an important direction for future work. However, such studies are inherently limited to specific combinations of imaging parameters, pulse sequences, and hardware, which vary substantially across scanner platforms and application-specific requirements. Given this variability, we encourage researchers to evaluate their own imaging setup as preliminary work prior to data collection. The contribution of this work is therefore to provide physics-based insight and practical guidance that can be adapted to individual imaging contexts, rather than to prescribe a single optimized acquisition strategy.

To our knowledge, no validation studies have systematically assessed off-isocentre distortions in muscle diffusion MRI. Future studies should therefore include direct validation of the considerations presented here to further strengthen their applicability and impact. However, existing validation approaches, whether using animal ^5, 53^, human ex vivo ^25^, or in vivo data ^35^, each face limitations. Animal and cadaver studies are costly and may not align with human in vivo properties, and obtaining 3D muscle fibre data through dissection is challenging: Some studies used the average pennation angle of only one ^25^ or five fibres ^5^, while others digitized around 100 fascicles manually ^53^. In vivo validation performed using 3D ultrasound imaging ^35^ also presents limitations, although volumetric fibre reconstruction methods are under development ^54^. As a result, the direct anatomical validation of the tractography-derived fibre reconstructions obtained in vivo remains challenging. In this context, agreement of fibre reconstructions across overlapping slices and continuity of fibre reconstructions across adjacent stacks should be interpreted as a form of internal consistency or verification rather than direct validation against anatomical ground truth. While such agreements do not establish anatomical accuracy, they provide a practical means of identifying acquisition- or reconstruction-related inconsistencies. Therefore, we suggest acquiring stacks with overlapping slices to assess consistency of fibre reconstructions across stack boundaries, and, if necessary, adjusting image settings based on our practical considerations presented here. In principle, increasing the number of overlapping slices may improve the robustness of this internal consistency assessment, but at the cost of longer acquisition times and increasing redundancy. The present study was not designed to determine an optimal number of overlapping slices, and the appropriate degree of overlap is expected to depend on factors such as stack length, anatomical coverage, scanner-specific characteristics, and other acquisition settings; however, if agreement across a small number of overlapping slices is already satisfactory, additional overlap is unlikely to provide further benefit. However, it should be noted that agreement in overlapping slices is a useful internal quality-control tool from subject-specific in vivo data, but not a substitute for thorough anatomical validation of geometric calibration, e.g., using phantoms.

As muscle diffusion MRI has the capability of characterizing volumetric fibre architecture, it provides valuable information for the modelling of the transversely isotropic tissue behaviour (e.g., ^7^). When applied to characterize muscle architecture changes in patient cohorts (e.g., ^29, 55^), accurate and repeatable data acquisition becomes a basic prerequisite to derive clinically meaningful metrics. Therefore, it is critical to assess the quality of the 3D muscle reconstructions and identify pitfalls in muscle diffusion MRI that may compromise accuracy. This work addresses the underlying issues and provides recommendations to optimize acquisition settings and processing pipelines, facilitating more reliable fibre tracking and modelling of muscle function.

## Conclusion

This work highlights key pitfalls in skeletal muscle diffusion MRI and tractography-based muscle fibre reconstruction from multi-stack acquisitions and offers mitigation strategies. Our images illustrate that large FOV muscle diffusion MRI can be affected by off-isocentre distortions and potential inaccuracies in the diffusion tensor estimation. The observations presented here suggest that shorter stack lengths can help reduce visible off-isocentre distortions and improve continuity of fibre reconstructions across stacks; however, the optimal stack length is inherently scanner- and protocol-dependent, and these effects should be interpreted in the context of concurrently varying acquisition and processing factors. Agreement across overlapping stacks provides a practical verification of internal consistency but does not constitute validation of muscle fibre architecture against anatomical ground truth. Table 1 summarizes practical considerations for configuring muscle diffusion MRI protocols, providing physics-informed guidance for researchers implementing large FOV multi-stack protocols. This work contributes technical insight and practical guidance that may support broader use of diffusion MRI in musculoskeletal research, although further validation will be required to establish reconstruction accuracy.

**Table 1:** List of considerations for multi-stack diffusion magnetic resonance imaging of skeletal muscles. FOV: field of view; DT-EPI: diffusion tensor echo-planar imaging.

	Feature	Consideration
Before acquisition	Participant positioning	Align the region of interest with the magnet isocentre as best as possible, e.g., perform unilateral rather than bilateral scans
		Control joint position and pressure on muscle tissue
	Multi-stack acquisition	Divide a large FOV through-plane into multiple stacks
		Acquire stacks with overlapping slices between stacks
		Decide on the maximum stack length by assessing off-isocentre distortions (e.g., from overlapping slices) in pilot acquisitions
	Coil type	Choose coil type (e.g., soft coil) that covers FOV without the need for coil repositioning
		Check compatibility of coil type and image acceleration techniques (stable and sufficient coil sensitivity profiles)
During acquisition	Field inhomogeneity	Perform higher-order active shimming routine if available
		Apply scanner-specific correction methods if available, e.g., real-time centre frequency correction method
	Phase-encode direction for EPI sequences	Acquire images with both (i.e., reverse) phase-encode direction or choose phase-encode direction such that chemical fat shifts are not manifesting in the region of interest
	Fat suppression	Improve insufficient fat suppression (fat ghosting) by improving field homogeneity
		Choose a fat saturation method which is less sensitive to field inhomogeneities, especially when using large FOVs (e.g., bilateral scans)
After acquisition	Correction methods	For DT-EPI sequences, perform susceptibility-induced distortion correction from reverse phase-encode images
		Correct for eddy current-induced distortions and movement artefacts if required
	Image registration	Register different image modalities if distortions are present and apply rotation to diffusion directions
		Add empty slices at stack boundaries (zero-padding) to prevent void slices in original FOV after applying transformation matrices
	Concatenating stacks	Concatenate multi-stack acquisitions into a continuous volume by averaging overlapping slices and applying intensity scaling across stacks
		For rotated diffusion directions per stack, compute the diffusion tensor stack by stack and calculate synthetic DT-EPI images with a common set of diffusion gradient directions across stacks

## Methods

### Image acquisition

MRI images of the lower leg were obtained from five healthy volunteers (2 females, 3 males, aged 22–42 years, 164–182 cm height, 60–80 kg weight) after obtaining informed consent (approval 20CEN107 from the New Zealand Health and Disability Ethics Committee) using a 3T clinical scanner (GE SIGNA Premier, GE Healthcare, Chicago, IL, USA). All methods were performed in accordance with the relevant guidelines and regulations, including the Declaration of Helsinki.

**Fig. 7:**
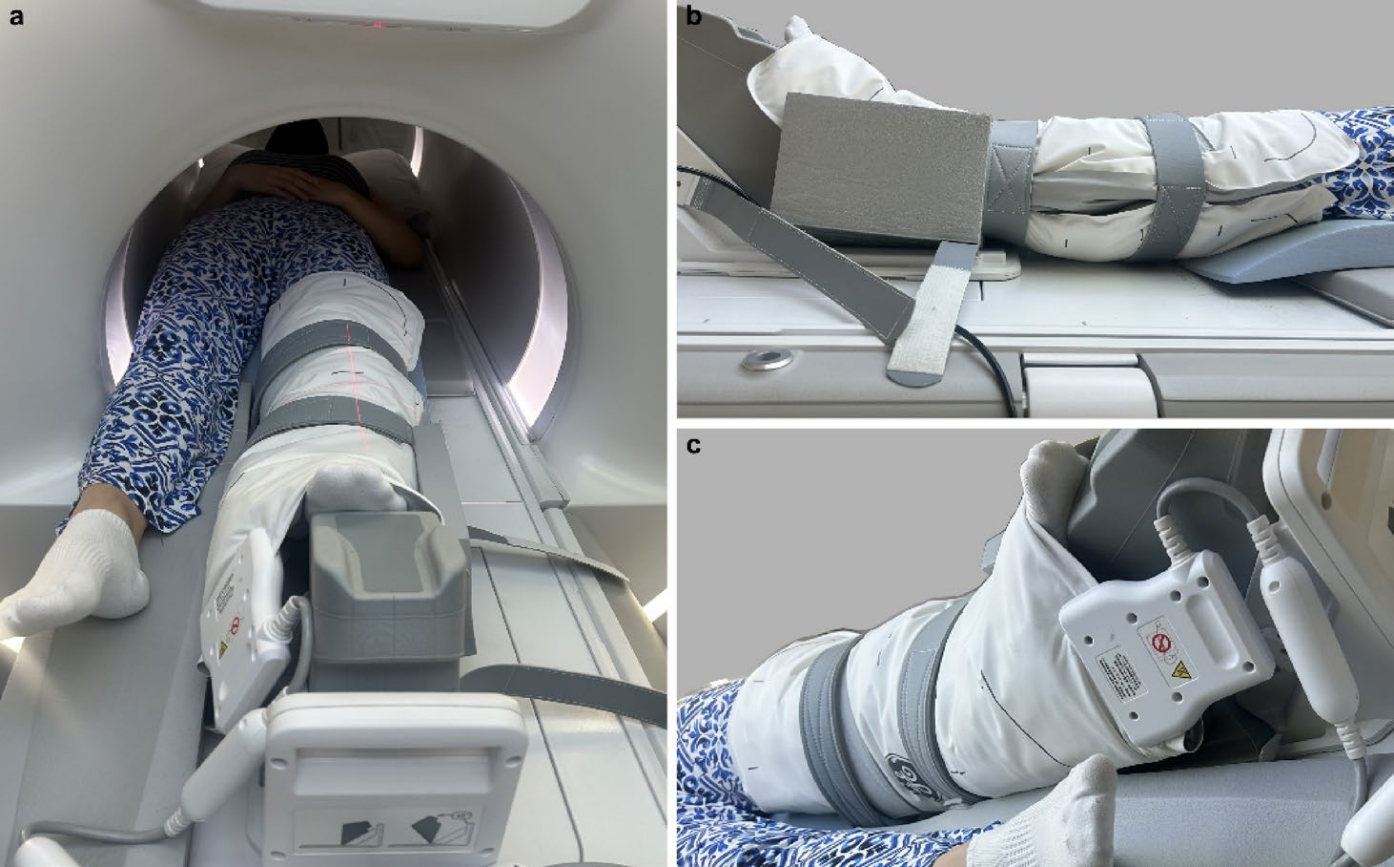
Participant positioned on the scanner table for a unilateral lower limb scan. The left leg of the participant was centred with the scanner axis using the scanner laser (**a**). The heel was positioned in a U-shaped cushion to support the ankle, and a cushion was placed underneath the thigh to elevate the calf from the scanner table (**b**). A flexible 30-channel coil was tightly wrapped around the entire lower limb and secured with two straps (**c**).

Axial images were acquired unilaterally or bilaterally using three sequences (Supplementary Table S1): FSE, 3D-GRE, and single-shot DT-EPI. A 30-channel AIR™ Anterior Array Coil, and for bilateral scans in combination with a 60-channel posterior array, was used. Participants were positioned supine, with the ankle and thigh elevated using cushions to prevent soft tissue compression, and entered the scanner bore in head-first-supine orientation (Fig. 7). The ankle was in a neutral position, and the knee was slightly flexed.

### Image processing

Scanner specific correction methods such as higher-order shimming routines or distortion correction are indicated when applied in Supplementary Table S1. Images were converted from DICOM to NIfTI format using *dcm2niiX* (version v1.0.20250505 ^56^, . All post-processing of DT-EPI images was performed for each stack separately. Using the FMRIB Software Library (*FSL*, version 6.0.7.19 ^57^, susceptibility-induced distortions were corrected from reverse phase-encode images using the *topup* function ^45^ when available (i.e., for scan ID 7 A, 7B, Supplementary Table S1). Motion and eddy current distortions were corrected in all DT-EPI images using the *eddy* function ^21^. An empty slice was concatenated to each end of the image stack (‘zero-padding’) prior to applying the eddy and motion correction and subsequently removed from the unwarped images using *MRTrix3* (version 3.0.4 ^58^, . A lower limb segmentation mask was used for the eddy and motion correction ^21^. The diffusion tensor and FA maps were calculated from the post-processed DT-EPI images for each stack using the *dtifit* function in *FSL (*version 6.0.7.19 ^57^, .

### Segmentation

Segmentation was performed by a trained musculoskeletal researcher (M.Z.) using *3D Slicer* (version 5.10 ^59^, , and segmentation masks were exported in compressed NIfTI format. Limb segmentation masks, i.e., masks that include all structures of the limb and exclude any background noise, were generated from FSE or 3D-GRE images in a semi-automatic manner. Using the threshold tool, a segmentation mask was generated by selecting (i) a lower threshold that differentiated noise and image intensities from the limb, and (ii) the maximum intensity of the image as the upper threshold. The operator then inspected every slice and ensured that the mask had no holes, isolated pixel groups (‘islands’), and a smooth boundary that represented the skin.

Muscle segmentation masks were generated manually on the FSE images by outlining the visible muscle boundaries of the gastrocnemius medialis, gastrocnemius lateralis, or soleus muscle. To evaluate geometric distortions at the stack periphery, muscle masks were also segmented from the DT-EPI images without diffusion-weighting for the overlapping slices between adjacent stacks. For DT-EPI images with reversed phase-encode direction, this was performed for both phase-encode directions separately.

### Image stack concatenation

FSE and DT-EPI multi-stack acquisitions were concatenated to a continuous volume in *MATLAB* (version R2024b, The MathWorks Inc., Natick, MA, USA). The motion and eddy current correction applied to the DT-EPI stacks during image post-processing resulted in stack-specific diffusion gradient directions. To concatenate the DT-EPI image stacks using a common set of diffusion gradient directions, synthetic DT-EPI images were regenerated from the post-processed DT-EPI images for each stack using the Stejskal-Tanner equation:

1 $$\normalsize \normalsize \:S={S}_{0}\cdot\:{e}^{-b{{u}}^{T}{D}|{u}|}$$

with $\:S$: synthetic diffusion MRI signal; $\:{S}_{0}$: average diffusion MRI signal without diffusion gradient applied; $\:b$: diffusion gradient strength (b-value); $\:\boldsymbol{u}$: unit diffusion gradient direction vector; $\:\boldsymbol{D}$: diffusion tensor in a quadratic form.

Calculations were restricted to the limb segmentation mask to avoid recalculating background noise. Diffusion gradient strength and diffusion gradient direction were chosen as the original acquisition settings. For DT-EPI images acquired with reversed phase-encode direction, this was performed for each phase-encode direction separately. Subsequently, the images acquired with reversed phase-encode directions were combined into a single image matrix. Note that any bias or instability introduced during tensor fitting may propagate into the regenerated synthetic DT-EPI images.

Synthetic DT-EPI images and FSE images were concatenated in pairs of two adjacent stacks: After concatenating two stacks, the resulting volume was concatenated sequentially with the subsequent stack. Stack concatenation included averaging of overlapping slices and scaling of image intensities.

Averaging overlapping slices: The slices in the overlapping area of two subsequent stacks were calculated as the weighted average, assigning greater weights to slices closer to the centre of the stack. For $\:N$ overlapping slices in each stack, the weight *w* for a slice $\:i\in\:\{\mathrm{1,2},\dots\:,N\}$ with $\:i$ increasing from the stack periphery towards the stack centre was defined as.

2 $$\:{w}_{i}=\:\frac{i-1}{N-1}\mathrm{\:with\:}i=\{\mathrm{1,2},\dots\:,\:N\}$$

Intensity scaling: When concatenating two adjacent stacks, the image intensities of the second stack were scaled by an intensity ratio obtained from the overlapping slices of the two stacks. Let $\:{I}_{1}\left(\mathbf{x}\right)$ and $\:{I}_{2}\left(\mathbf{x}\right)$ denote the image intensities of the overlapping slices from the first and second stack, respectively, at pixel location $\:\mathbf{x}$ within the overlapping region $\:{\Omega\:}$. The intensity ratio $\:s$ was computed as the mean of the pixel-wise intensity ratios across the overlapping region.

3 $$\:s=\frac{1}{\left|{\Omega\:}\right|}\sum\:_{\boldsymbol{x}\in\:{\Omega\:}}\frac{{I}_{1}\left(\boldsymbol{x}\right)}{{I}_{2}\left(\boldsymbol{x}\right)}\:$$

The image intensities of the second stack were then scaled by this factor $\:s$. For the diffusion tensor echo-planar images, the intensity ratio was calculated from the non-diffusion-weighted images.

Muscle segmentation masks were manually regenerated for the concatenated FSE images in the overlapping area.

### Tractography

Deterministic tractography was performed in *MRTrix3 *^58^ using the *tckgen* command ^60^. The muscle segmentation mask of the gastrocnemius medialis, gastrocnemius lateralis, or soleus muscle was used as mask and seed points with the following settings: step size: 0.1x voxel size; maximum angle between successive steps: 60°; minimum length of any track: 5x voxel size; maximum length of any track: 100x voxel size; tensor FA cutoff for terminating tracks: 0.1. The desired number of streamlines to be selected was 1000 for all images except for scan ID 5 A, distal stack (Supplementary Table S1). Here, only 500 streamlines were selected.

### Quantitative analyses

For each voxel within the limb segmentation mask, the 3D coordinates $\:(x,y,z)$ were computed in scanner space and converted to physical coordinates using the image affine transform. The $\:x$- and $\:y$- coordinates corresponded to the in-plane voxel positions relative to the magnet isocentre, and the $\:z$-coordinate was derived from the slice location, assuming the stack was centred at the magnet isocentre. The radial distance $\:R$ from the magnet isocentre of each voxel was then calculated as:

4 $$\:R=\sqrt{{x}^{2}+{y}^{2}+{z}^{2}}$$

The maximum radial distance within the limb mask was subsequently extracted as $\:{R}_{max}=\mathrm{m}\mathrm{a}\mathrm{x}\left(R\right)$.

Similarity of gastrocnemii muscle masks obtained from overlapping slices in multi-stack acquisitions was quantified using the DSC, defined as

5 $$\:DSC=\frac{2\left|A\bigcap\:B\right|}{\left|A\right|+\left|B\right|}$$

where $\:A$ and $\:B$ denote the voxel sets of the two segmentation masks obtained from overlapping slices of adjacent stacks.

Moreover, FA maps of overlapping slices were subtracted to visualize a FA difference map, and the RMSE of FA values was computed voxel-wise between overlapping slices and averaged over all voxels within the gastrocnemii muscle mask.

Note that DSC of segmentation masks and RMSE of FA maps are reported descriptively to support the visual observations presented in the illustrations. Formal statistical inference was not undertaken, as the quantitative analyses were performed on a set of exemplary images acquired with heterogeneous imaging protocols.

## Supplementary Information

Below is the link to the electronic supplementary material.

Supplementary Material 1

## Data Availability

The data that support the findings of this study are available from the corresponding author upon reasonable request.
