# Supplementary material for: Overcoming pitfalls in multi-stack diffusion MRI for tractography reconstruction of skeletal muscles
Source: Sci Rep. 2026 Jul 22;16:22938. doi: 10.1038/s41598-026-63269-6 (PMC13392244; doi:10.1038/s41598-026-63269-6)
Supplement: Supplementary file 1 — Supplementary Material 1 [file 41598_2026_63269_MOESM1_ESM.docx]

**Supplementary Materials**

For the manuscript entitled **“Overcoming pitfalls in multi-stack diffusion MRI for tractography reconstruction of skeletal muscles”** by *Manuela Zimmer, Geoffrey Handsfield, Paul Condron, Samantha Holdsworth, Flavio Dell’Acqua, and Filiz Ateş.*


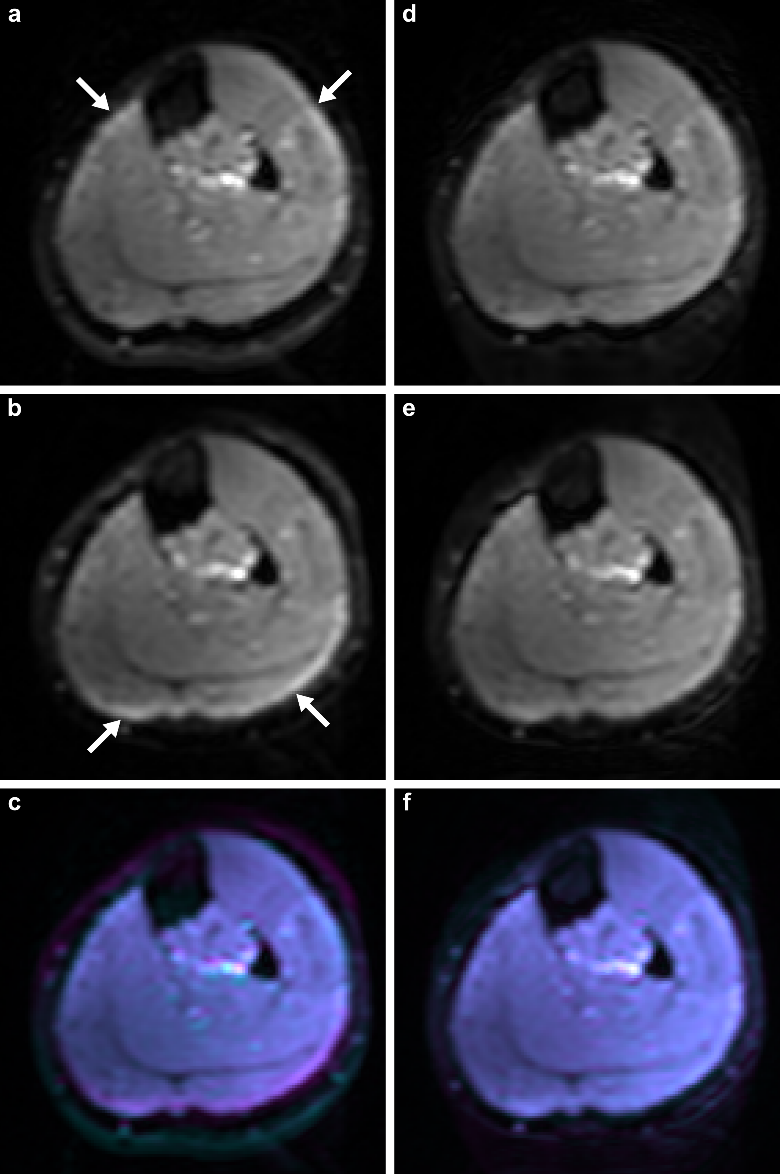


Figure S1. Susceptibility-induced distortion correction.

Axial echo-planar images (no diffusion-weighting) with phase-encode direction anterior-posterior (a) and posterior-anterior (b) demonstrate susceptibility-induced distortions and chemical fat shift artefacts (arrows). Susceptibility-induced distortion correction successfully unwarped the images (d, e), as illustrated by the overlay of the images with reverse phase-encode directions before (c) and after (f) the correction. The overlay visualizes the images with phase-encode direction anterior-posterior in cyan and posterior-anterior in magenta. Images were taken from a 28-year-old woman (body weight and height: 75 kg, 175 cm). Image settings are listed in Table 2 (ID 7A and 7B).


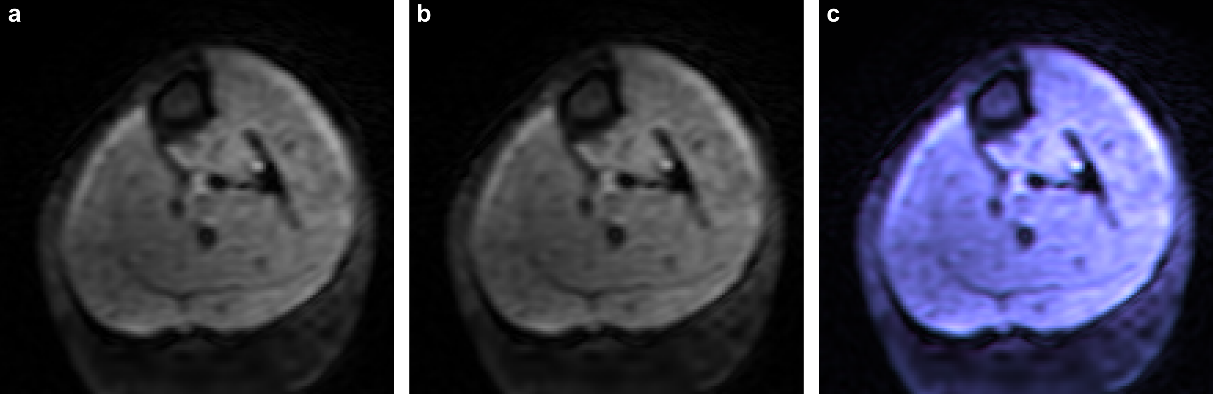


Figure S2. Motion and eddy current correction.

Axial diffusion-tensor echo-planar images (b-value = 500 s·mm^-2^) before (a) and after (b) motion and eddy current correction. The image overlay (c) does not indicate differences between the images, suggesting that motion- and eddy current-induced artefacts were negligible. The overlay visualized the images before correction in cyan and after correction in magenta. Images were taken from a 28-year-old woman (body weight and height: 75 kg, 175 cm). Image settings are listed in Table 2 (ID 7A).

Table S1. Image acquisition settings for the illustrated images.

| **Scan ID** | **Leg** | **Sequence** | **TR (ms)** | **TE (ms)** | **Acq. matrix size** | **Recon. matrix size** | **Voxel dimensions (mm)** | **Slice thickness (mm)** | **Stack length (cm)** | **Slices per stack** | **Slices overlap between stacks** | **Diffusion gradient dir.** | **PE dir.** | **Fat suppression method** | **Scan duration (min:s)** |
| --- | --- | --- | --- | --- | --- | --- | --- | --- | --- | --- | --- | --- | --- | --- | --- |
| 1A | Both | DT-EPI | 5780 | 45.8 | 150×90 | 256×256 | 1.64×1.64×4 | 4 | 26.4 | 66 | - | 15 | PA | Chem. fat sat. | 3:28 |
| 1B | Both | 3D-GRE | 5.8 | 2.7 | 500×500 | 1024×1024 | 0.44×0.44×1 | 2 | 26.4 | 264 | - | - | - | - | 0:53 |
| 2A | Both | DT-EPI | 11385 | 45.8 | 150×90 | 256×256 | 1.64×1.64×4 | 4 | 52.0 | 130 | - | 15 | PA | Chem. fat sat. | 6:50 |
| 2B | Both | 3D-GRE | 5.7 | 2.7 | 500×500 | 1024×1024 | 0.44×0.44×1 | 2 | 54.0 | 540 | - | - | - | - | 1:47 |
| 3 | Both | DT-EPI* | 3000 | 52.3 | 160×90 | 256×256 | 1.64×1.64×4 | 4 | 13.6 | 34 | 4 | 30 | PA | Chem. fat sat. wider bandwidth | 1:44 |
| 4 | Both | DT-EPI^†^ | 2351 | 46.5 | 150×90 | 256×256 | 1.64×1.64×4 | 4 | 22.4 | 56 | - | 15 | AP | Slice-selective gradient reversal | 1:25 |
| 5A | Left | DT-EPI^†^ | 4000 | 49.2 | 120×80 | 256×256 | 1.02×1.02×4 | 4 | 24.0 | 60 | 7 | 30 | AP | Hybrid chem. fat sat. & Inv. recov. | 4:24 |
| 5B | Left | FSE | 664 | 8.6 | 500×500 | 1024×1024 | 0.25×0.25×4 | 4 | 24.0 | 60 | 7 | - | - | - | 2:16 |
| 6A | Left | DT-EPI | 6082 | 46.8 | 80×64 | 256×256 | 0.86×0.86×4 | 4 | 16.8 | 42 | 4 | 30 | PA | Hybrid chem. fat sat. & Inv. recov. | 6:41 |
| 6B | Left | FSE | 710 | 8.9 | 500×500 | 1024×1024 | 0.21×0.21×4 | 4 | 16.8 | 42 | 4 | - | - | - | 1:46 |
| 7A | Left | DT-EPI^*,⁑^ | 4517 | 50.9 | 80×96 | 256×256 | 1.02×1.02×4 | 4 | 15.2 | 38 | 4 | 6 | AP | Chem. fat sat. wider bandwidth | 1:23 |
| 7B | Left | DT-EPI^*,⁑^ | 4801 | 50.9 | 80×96 | 256×256 | 1.02×1.02×4 | 4 | 15.2 | 38 | 4 | 15 | PA | Chem. fat sat. wider bandwidth | 2:55 |
| 7C | Left | FSE^*,⁑^ | 625 | 8.6 | 500×500 | 1024×1024 | 0.25×0.25×4 | 4 | 15.2 | 38 | 4 | - | - | - | 1:35 |

DT-EPI: diffusion tensor echo-planar imaging, with b-value of 500 s∙mm^-2^ and two excitations; FSE: fast-spin echo; 3D-GRE: 3-dimensional gradient echo; TR: repetition time; TE: echo time; Acq.: Acquisition; Recon.: Reconstruction; Diffusion gradient dir.: Diffusion gradient directions; PE dir: Phase-encode direction; PA: posterior-anterior; AP: anterior-posterior; Chem. fat. sat.: chemical fat saturation; Inv. recov.: inversion recovery; *Real-time B_0_ frequency correction was used; ⁑ Higher-order shimming preceded the scan; †Simultaneous multi-slice acquisition (with multi slice factor 2) and scanner distortion correction were performed.
